# Screening for obstructive sleep apnea before coronary angiography

**DOI:** 10.1111/crj.13556

**Published:** 2022-11-20

**Authors:** Guo Pei, Qiong Ou, Yongchi Chen, Yanxia Xu, Jiaoying Tan

**Affiliations:** ^1^ Department of Sleep Center, Guangdong Provincial People's Hospital, Guangdong Academy of Medical Sciences Guangdong Provincial Geriatrics Institute Guangzhou China; ^2^ School of Medicine South China University of Technology Guangzhou China; ^3^ Geriatric Cardiology, Guangdong Provincial People's Hospital, Guangdong Academy of Medical Sciences Guangdong Provincial Geriatrics Institute Guangzhou China

**Keywords:** coronary angiography, coronary disease, early screening, obstructive sleep apnea, sleep monitoring

## Abstract

**Introduction:**

The prevalence of obstructive sleep apnea (OSA) in patients with suspected coronary heart disease (CHD) is yet to be clarified. This study aimed to investigate the prevalence of OSA before coronary angiography (CAG).

**Methods:**

We retrospectively evaluated patients with suspected CHD admitted to the Department of Geriatric Cardiology of our hospital between July 2019 and July 2021. OSA was screened using the level III home sleep apnea test before CAG. The prevalence of OSA was then compared between the CHD and non‐CHD groups. CHD severity was determined using the Gensini score of CAG results, and OSA severity was graded using the apnea‐hypopnea index (AHI).

**Results:**

Among the 327 patients, 211 had CHD. In total, 264 patients were diagnosed with OSA (80.7%) (184 patients, CHD group [87.2%]; 80 patients, non‐CHD group [69.0%]). The CHD group had a significantly higher prevalence of OSA (*P* < 0.01) and higher AHI (CHD group 18.76 ± 14.94, non‐CHD group 11.56 ± 10.67, *P* < 0.01). The Gensini score was positively correlated with OSA severity in patients with CHD, and AHI ≥ 20 was a risk factor for CHD (odds ratio: 1.961, 95% confidence interval: 1.065–3.608, *P* < 0.05).

**Conclusion:**

OSA screening before CAG revealed a higher prevalence in CHD patients than in non‐CHD patients. The degree of coronary artery obstruction is positively correlated with AHI, and AHI ≥ 20 is a risk factor for CHD. Therefore, attention should be paid to OSA screening and management before CAG in patients with suspected CHD.

## Graphical abstract

OSA screening for patients with suspected coronary heart disease before CAG showed that the prevalence of OSA in CHD patients was higher than that in non‐CHD patients. AHI ≥ 20 is a risk factor for CHD. Therefore, OSA screening and management should be paid attention to before CAG in patients with suspected CHD.

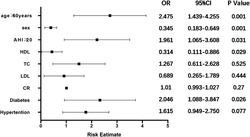

## 1 INTRODUCTION

Obstructive sleep apnea (OSA) or hypoventilation is a common sleep‐related respiratory disorder caused by repeated airway collapse during sleep.^1^ It is characterized by snoring, repeated hypoxemia at night and daytime sleepiness. The series of pathophysiological changes that occur in the body lead to functional and organic changes in the vital organs.^2^ Epidemiological studies have shown that OSA occurs in 2–4% of adults and in 4.63% among those aged 30 years. Particularly, the prevalence of OSA can be as high as 30–50% among patients with hypertension and coronary heart disease (CHD).^3^ OSA could be possibly associated with early atherosclerosis as it promotes cellular changes at the endothelial level, which may contribute to atherosclerosis.^4^ OSA can also increase the occurrence of CHD and the morbidity and mortality associated with CHD and other cardiovascular diseases. OSA is a novel cardiovascular risk factor that shares many risk factors and comorbidities with cardiovascular diseases, including obesity, masculinity, advanced age, metabolic syndrome and hypertension.^5^ However, few studies have clarified the prevalence of OSA in patients with suspected CHD. Thus, this study aimed to analyse the prevalence of OSA before coronary angiography (CAG) in patients with suspected CHD.

## 2 MATERIALS AND METHODS

### 2.1 Study design and subjects

This retrospective study evaluated 336 patients (215 men and 121 women) aged 32–90 (60.38 ± 10.813) years who were admitted to the Department of Geriatric Cardiology of Guangdong Provincial People's Hospital for suspected CHD and underwent level III portable home sleep apnea test (HST) for snoring between July 2019 and July 2021. All patients underwent CAG because of a suspected CHD for the first time. The exclusion criteria were as follows: (1) left ventricular ejection fraction <40%; (2) central and mixed sleep apnea; (3) history of upper airway surgery or current continuous positive pressure ventilation (CPAP) therapy and (4) within 3 months use of oral sedative sleeping pills.

This study was approved by the ethics committee of Guangdong Provincial People's Hospital (No. GDREC2019757H).

### 2.2 General information and laboratory examination

Basic patient data, including sex, age, body mass index (BMI), personal history, family history and complications, were collected. On day 2 of admission, fasting blood samples were collected, and blood cell analysis, liver function, renal function, blood lipid and blood sugar levels were measured before CAG was performed.

### 2.3 Sleep apnea monitoring

All patients were monitored for the sleep apnea‐hypopnea index (AHI) and oxygen saturation (SpO_2_) using a level III HST (Alice NightOne, PHILIPS) one night before angiography. The AHI was defined as the average number of apnea and hypopnea events per hour. OSA was diagnosed according to symptoms of sleep apnea, such as habitual snoring, interrupted breathing or awakening from sleep due to holding breath, and apnea or hypopnea occurring more than five times per hour, with a minimum SpO_2_ at night as a reference, following the American Academy of Sleep Medicine Clinical Practice Guideline^6^ for the diagnosis and treatment of OSA. OSA severity was classified as follows: mild, AHI ≥ 5, <15 times/h; moderate, AHI > 15, ≤30 times/h; and severe, AHI ≥ 30 times/h. Mild hypoxemia was defined as the lowest SpO_2_ at night being 85–90%; moderate, 80–84%; and severe, <80%. The CHD group was divided into a simple CHD group (AHI < 5 times/h), mild OSA group (5 ≤ AHI < 15 times/h), moderate OSA group (15 ≤ AHI < 30 times/h) and severe OSA group (AHI ≥ 30 times/h).

### 2.4 Coronary angiography

CAG was performed by at least an experienced deputy director of the Department of Cardiology of our hospital. The patients were diagnosed with CHD when lumen stenosis of one or more major vessels was ≥50%, following the American College of Cardiology/American Heart Association guidelines. The patients were then divided into the CHD and non‐CHD groups according to the CAG results. According to the latest segmented evaluation standard of the coronary artery image score,^7^ the Gensini scoring system was adopted to quantitatively evaluate the degree of stenosis of each coronary artery. The stenosis degree was measured at the most severe location using the following parameters: stenosis diameter <25% was counted as 1 point; ≥25% to <50%, 2 points; ≥50% to <75%, 4 points; ≥75% to <90%, 8 points; ≥90% to <99%, 16 points; and ≥99%, 32 points.

The above score was multiplied by the corresponding coefficient according to different coronary artery branches: left main artery disease: score ×5; lesions of left anterior descending branch: proximal ×2.5, middle ×1.5; pathological changes of diagonal branches: D1 ×1, D2 ×0.5; lesions of left circumflex branch: proximal ×2.5, distal ×1; posterior descending branch ×1; posterior ramus ×0.5; right coronary artery lesion: proximal, middle, distal and posterior descending branches all ×1. The total score for each lesion branch was the total score for the degree of coronary artery stenosis. The score when the main left coronary artery was completely occluded was defined as the highest score (32 × 5 = 160), and the score without a coronary artery lesion was defined as 0. Coronary artery scores were divided into three grades: normal (0), grade 1 (1–80) and grade 2 (81–160).

### 2.5 Statistical analysis

Normally distributed measurement data are expressed as mean ± standard deviation (^−^x ± s) and were compared between groups using the *t* test. Meanwhile, enumeration data are expressed as rate (%) and were compared between groups using the χ^2^ test. Pearson's test was used to determine the correlation between variables. Risk factors for CHD were analysed using logistic regression analyses. All statistical analyses were performed using SPSS 20.0 software (IBM SPSS 20.0, Armonk, NY, US). Statistical significance was set at *P* < 0.05.

## 3 RESULTS

### 3.1 Patient characteristics

In total, 327 patients were evaluated. Among them, 211 (64.53%) and 116 (35.47%) belonged to the CHD and non‐CHD groups, respectively. There were no significant between‐group differences in BMI and proportion of patients complaining of chest tightness, chest pain or shortness of breath between the two groups (all *P* > 0.05). The CHD group was older (*P* < 0.01) and included a higher proportion of men and patients with chief complaints of palpitations, hypertension and diabetes (all *P* < 0.01). The patient characteristics are shown in Table 1.

**TABLE 1:** Baseline patient characteristics

	CHD group (*n* = 211)	Non‐CHD group (*n* = 116)	*P*
Age (y), mean ± SD	61.79 ± 11.15	58.12 ± 9.98	0.003
Sex, *n* (%)			<0.01
Male	153 (72.5%)	57 (49.1%)	
Female	58 (27.5)	59 (50.9%)	
BMI (kg/m^2^), mean ± SD	24.45 ± 3.68	24.24 ± 2.92	0.601
Chest distress, *n* (%)	175 (82.9%)	91 (78.4%)	0.319
Chest pain, *n* (%)	127 (60.2%)	62 (53.4%)	0.238
Heart palpitations, *n* (%)	13 (6.2%)	18 (15.5%)	0.006
Shortness of breath, *n* (%)	24 (11.4%)	17 (14.7%)	0.391
Hypertension, *n* (%)	134 (63.5%)	56 (48.3%)	0.008
Diabetes, *n* (%)	75 (35.5%)	21 (18.1%)	0.001
Hyperlipidemia, *n* (%)	65 (30.8%)	47 (40.5%)	0.082
Hyperuricemia, *n* (%)	43 (20.4%)	31 (26.7%)	0.19
AHI (events/h), mean ± SD	18.76 ± 14.94	11.56 ± 10.67	<0.01
AHI ≥ 20, *n* (%)	73 (34.6%)	19 (16.4%)	<0.01
Average saturation (%), mean ± SD	94.15 ± 1.58	94.53 ± 1.34	0.026
Minimum saturation (%), mean ± SD	83.93 ± 6.35	85.62 ± 6.80	0.026
Time of saturation<90% (min), mean ± SD	21.86 ± 52.60	12.28 ± 36.42	0.054
Creatinine (mmol/L), mean ± SD	76.85 ± 18.54	70.31 ± 15.65	0.001
Total cholesterol (mmol/L), mean ± SD	4.68 ± 1.38	5.09 ± 1.34	0.01
Triglycerides (mmol/L), mean ± SD	1.60 ± 0.97	1.65 ± 1.14	0.659
LDL cholesterol (mmol/L), mean ± SD	2.92 ± 1.00	3.25 ± 0.99	0.006
HDL cholesterol (mmol/L), mean ± SD	1.18 ± 0.26	1.29 ± 0.36	0.001
LVEF (%), mean ± SD	63.38 ± 6.43	64.11 ± 6.42	0.327
OSA, n (%)	184 (87.2%)	80 (69.0%)	<0.01

Abbreviations: AHI, apnea‐hypopnea index; BMI, body mass index; CHD, coronary heart disease; HDL, high‐density lipoprotein; LDL, low‐density lipoprotein; LVEF, left ventricular ejection fraction; OSA, obstructive sleep apnea.

### 3.2 Comparison of laboratory and sleep respiration monitoring indicators

There were no significant differences in the prevalence of hyperlipidemia, prevalence of hyperuricemia, triglyceride level and left ventricular ejection fraction between the CHD and non‐CHD groups (*P* > 0.05). However, creatinine levels were higher in the CHD group (*P* < 0.01), while the levels of total cholesterol, low‐density lipoprotein and high‐density lipoprotein were higher in the non‐CHD group (*P* < 0.01) (Table 1).

A total of 264 patients were diagnosed with OSA: 184 (87.2%) patients in the CHD group and 80 cases (69.0%) in the non‐CHD group, with the prevalence of OSA being significantly higher in the CHD group (*P* < 0.01). Meanwhile, the results of sleep respiration monitoring showed that there was no statistically significant difference in the time of blood oxygen <90% between the CHD and non‐CHD groups (*P* > 0.05). AHI was higher in the CHD group than in the non‐CHD group (P < 0.01), and both mean and minimum blood oxygen levels were higher in the non‐CHD group than in the CHD group (*P* < 0.01) (Table 1).

### 3.3 Comparison of CAG results among the severe OSA, moderate OSA, mild OSA and simple CHD groups

There were 27, 77, 63 and 44 patients in the simple CHD, mild OSA, moderate OSA and severe OSA groups, respectively. In these groups, the rates of one coronary artery involvement were 20.5%, 30.2%, 42.9% and 33.3%; two coronary arteries, 38.6%, 30.2%, 20.8% and 18.6%; and three coronary arteries, 40.9%, 39.7%, 36.4% and 48.1%, respectively. The coronary artery involvement rate did not differ according to OSA severity (*P* = 0.149). The average Gensini scores of the severe OSA, moderate OSA, mild OSA and simple CHD groups were 38.18 ± 34.53 points, 24.67 ± 20.41 points, 24.91 ± 23.09 points and 26.15 ± 19.47 points, respectively. The Gensini score was significantly higher in the severe OSA group than in the other groups (*P* = 0.022) (Table 2).

**TABLE 2:** Comparison of CAG results among OSA groups

CHD group	*n*	Number of vessels involved			Gensini score
		1	2	3	
Complicated with severe OSA	44	9 (20.5%)	17 (38.6%)	18 (40.9%)	38.18 ± 34.532
Complicated with moderate OSA	63	19 (30.2%)	19 (30.2%)	25 (39.7%)	24.67 ± 20.413
Complicated with mild OSA	77	33 (42.9%)	16 (20.8%)	28 (36.4%)	24.91 ± 23.094
Complicated with no OSA	27	9 (33.3%)	5 (18.6%)	13 (48.1%)	26.15 ± 19.475
*X* ^2^/*F*	6				3.289
*P*	0.149				0.022

Abbreviations: CHD, coronary heart disease; OSA, obstructive sleep apnea.

### 3.4 Risk factors of CHD

**FIGURE 1:**
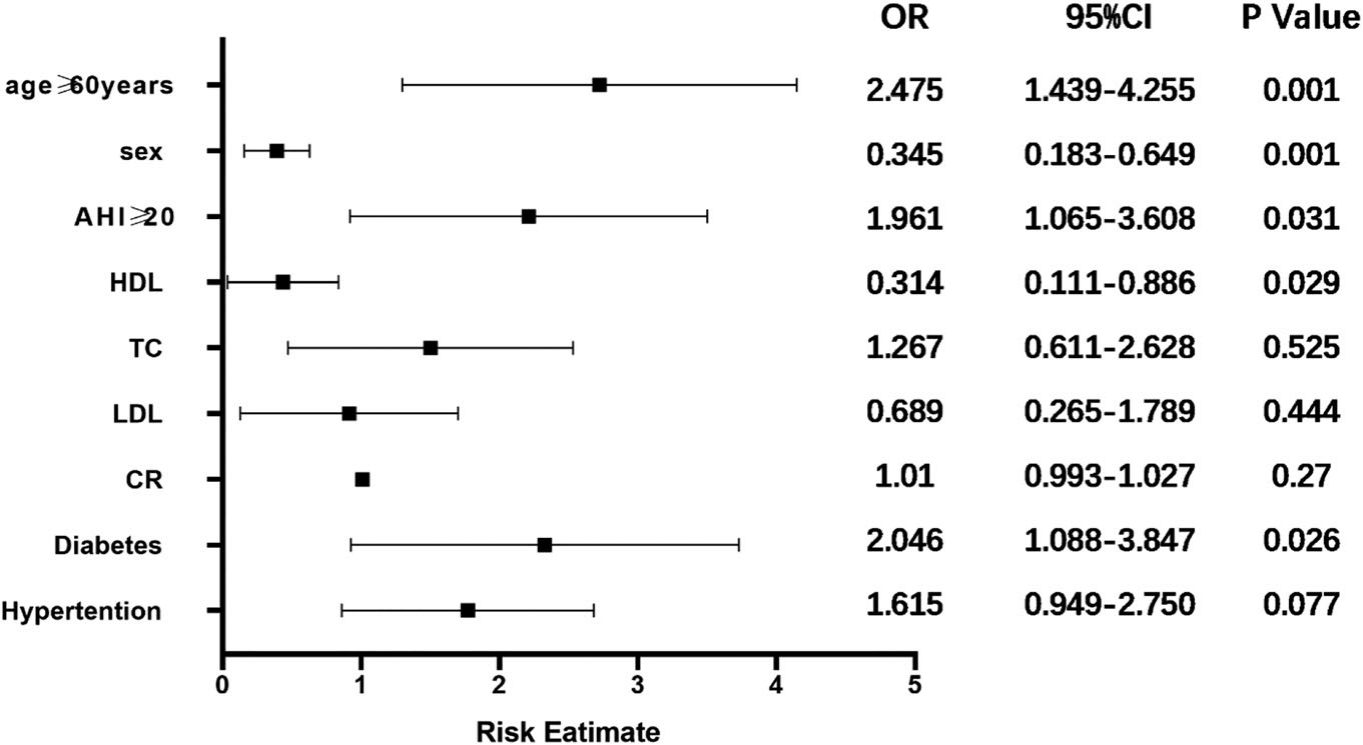
Forrest plot with predictors of coronary heart disease (CHD) in multivariate analysis. Values are expressed in odds ratio (OR) and confidence interval (CI) of 95%. AHI, apnea‐hypopnea index; HDL, high‐density lipoprotein; TC, total cholesterol; LDL, low‐density lipoprotein; CR, creatinine

Multivariate logistic regression analysis was performed with CHD as the dependent variable and sex, age, hypertension, diabetes, AHI, average blood oxygen, minimum blood oxygen and creatinine as independent variables. The CHD risk factors are shown in Table 3. Diabetes (odds ratio [OR]: 2.046, 95% confidence interval [CI]: 1.088–3.847), AHI ≥ 20 (OR, 1.961, 95% CI: 1.065–3.608), sex (OR: 0.345, 95% CI: 0.183–0.649) and age ≥60 years (OR: 2.475, 95% CI: 1.439–4.255) were significantly positively correlated with CHD. High‐density lipoprotein (OR: 0.314, 95% CI: 0.111–0.886) was a protective factor against CHD (Figure 1).

**TABLE 3:** Multivariate logistic analysis of CHD‐related risk factors

Characteristic	OR	95% CI	*P*
Hypertension	1.615	0.949–2.750	0.077
Diabetes	2.046	1.088–3.847	0.026
Creatinine	1.01	0.993–1.027	0.27
Total cholesterol	1.267	0.611–2.628	0.525
LDL	0.689	0.265–1.789	0.444
HDL	0.314	0.111–0.886	0.029
AHI ≥ 20	1.961	1.065–3.608	0.031
Sex	0.345	0.183–0.649	0.001
Age ≥ 60 years	2.475	1.439–4.255	0.001

Abbreviations: AHI, apnea‐hypopnea index; CHD, coronary heart disease; CI, confidence interval; HDL, high‐density lipoprotein; LDL, low‐density lipoprotein; OR, odds ratio.

## 4 DISCUSSION

In this study, OSA screening before CAG in patients with suspected CHD confirmed the high prevalence of OSA in patients with CHD and the independent association between OSA and CHD severity. Moreover, CAG results showed that normal patients may also have undiagnosed OSA. Collectively, these findings support the importance of screening for OSA before CAG in patients with suspected CHD.

OSA is a common chronic disease that is prevalent in 4% of adult men and 2% of adult women.^8^ The prevalence of OSA is more than two times higher in patients with CHD than in those without CHD, and CHD is more strongly correlated with sleep breathing disorder in men than in women.^9^ Our findings are consistent with previous evidence. The prevalence of OSA in patients with CHD was 87.2%, and the degree of coronary artery obstruction in patients with CHD increased with the severity of OSA. AHI ≥ 20 was a risk factor for CHD, consistent with both local and international findings.^10^, ^11^ In addition, the levels of total cholesterol and low‐density lipoprotein were higher in the non‐CHD group than in the CHD group, suggesting that OSA is associated with other factors of metabolic syndrome, such as hyperlipidemia, insulin resistance, and obesity.^12^, ^13^ There was no significant difference in the proportion of patients with chest distress, chest pain and shortness of breath as the chief complaints between the CHD and non‐CHD groups. This indicates that the chief complaints of patients with suspected CHD could not be used to accurately distinguish between CHD and OSA. Chest distress and other symptoms in the non‐CHD group were not caused by CHD but by OSA. The HST results also showed that there was no difference in the time of blood oxygen <90% between the two groups, indicating that chest tightness and other symptoms in the non‐CHD group might be caused by OSA hypoxia at night, which was mistaken by patients for symptoms of CHD.

The coronary vascular system can be assessed using either non‐invasive or invasive procedures. Among these, CAG has been established as a reliable method for assessing coronary arteries in patients at high risk of CHD.^14^ The Gensini score can accurately quantify coronary artery lesions; the higher the score, the more serious the stenosis. In this study, Gensini scores increased with OSA severity, but the incidence of coronary artery involvement did not differ according to OSA severity among the CHD patients. Studies using non‐invasive coronary computed tomography angiography have found that OSA patients have a higher proportion of multivessel involvement than non‐OSA patients. Further, OSA patients have more non‐calcified plaques or mixed plaques in the coronary arteries and a more severe degree of coronary artery stenosis.^15^ Thus, patients with OSA have more severe atherosclerosis than those without OSA.^16^

The current study also found that diabetes was positively correlated with CHD and that patients with diabetes had a 2.01 times higher risk of CHD than those without diabetes. Diabetes is an important independent risk factor for cardiovascular disease, which is in turn the most common cause of death in patients with diabetes.^17^ Patients with diabetes should be closely monitored for CHD. However, our research found that hypertension and lipid metabolism indices, such as creatinine and total cholesterol, were not significantly associated with the risk of CHD, which may be related to the limited number of cases studied.

Preoperative screening for OSA also reduces the incidence of postoperative complications. Studies have found that among high‐risk adults undergoing major noncardiac surgery, unrecognized severe OSA is significantly associated with an increased risk of cardiovascular complications 30 days postoperatively.^18^ In addition, OSA has adverse effects on the postoperative outcomes of patients undergoing cardiac surgery and increases the incidence of postoperative pneumonia.^19^ Therefore, it is necessary to improve our understanding of preoperative screening for OSA.

In this study, the prevalence of OSA in the non‐CHD group was significantly higher, which may be due to the following reasons: First, the selected patients were hospitalized for suspected CHD, and they generally complained of chest tightness, chest pain, palpitation, shortness of breath and other symptoms, which may be caused by CHD or OSA. In addition, the proportion of patients with admission symptoms of chest tightness, chest pain and shortness of breath in the non‐CHD group was not significantly different from that in the CHD group (*P* > 0.01), and the proportion of patients with admission symptoms of palpitation in the non‐CHD group was higher than that in the CHD group (*P* < 0.01). After CAG excluded CHD post‐admission, the symptoms of chest tightness, chest pain, palpitation and shortness of breath in non‐CHD patients may be caused by OSA. Second, the selected subjects were all from the elderly cardiovascular department, and their age was high, with an average of 60.38 years. Previous studies have shown that the prevalence of OSA increases with age, and it is generally high in the elderly population.^20^ Third, the elderly population selected in this study has a high prevalence of hypertension, and the prevalence of hypertension in the non‐CHD group was 48.3%. There is a significant overlap between OSA and systemic blood pressure.^21^ Some studies have also found that the risk of OSA in hypertensive patients is significantly higher than that in normotensive subjects,^22^, ^23^ which may also contribute to the significantly higher prevalence of OSA in the non‐CHD group in the present study.

This study had some limitations. First, this was a single‐centre retrospective study. Second, CPAP therapy and postoperative follow‐up were not performed in moderate and severe OSA patients in the CHD and non‐CHD groups. Third, the influence of OSA on the body is a process of chronic injury, and the time of OSA onset could not be accurately determined in this study. Fourth, we did not use a complete Epworth sleepiness scale to assess the degree of sleepiness in OSA patients. Therefore, it is necessary to further study the relationship between OSA and suspected CHD patients in the future.

In conclusion, this study not only confirmed the high prevalence of OSA in patients with CHD and the independent association between OSA and CHD but also found a high prevalence of OSA in patients with normal CAG. The chief complaints of chest distress and pain in patients with suspected CHD were not all caused by CHD, and some were probably caused by OSA. However, patients often mistake OSA for cardiovascular symptoms and miss the opportunity for OSA diagnosis and treatment after angiography results are normal. Thus, OSA screening should be performed before angiography in patients with suspected CHD to avoid missed diagnosis of OSA and provide treatment after angiography.

## CONFLICT OF INTEREST

The authors have no conflicts of interest to disclose.

## ETHICS STATEMENT

This study was approved by the ethics committee of Guangdong Provincial People's Hospital (No. GDREC2019757H). All subjects signed an informed consent form and agreed to participate in the study.

## AUTHOR CONTRIBUTIONS

All authors contributed to the study conception and design. Data collection was performed by Qiong Ou, Guo Pei, Jiaoying Tan and Yongchi Chen. Data collection and analysis were performed by Guo Pei. The first draft of the manuscript was written by Guo Pei, and all authors commented on previous versions of the manuscript. All authors read and approved the final manuscript.

## Data Availability

The data that support the findings of this study are available on request from the corresponding author.
